# MU variability in CBCT‐guided online adaptive radiation therapy

**DOI:** 10.1002/acm2.14440

**Published:** 2024-06-19

**Authors:** Sean Tanny, Olga M Dona Lemus, Joshua Wancura, Nicholas Sperling, Matthew Webster, Hyunuk Jung, Yuwei Zhou, Fiona Li, Jihyung Yoon, Alexander Podgorsak, Dandan Zheng

**Affiliations:** ^1^ Department of Radiation Oncology University of Rochester Medical Center New York New York USA; ^2^ Department of Radiation Oncology University of Toledo Medical Center Toledo Ohio USA

**Keywords:** adaptive radiotherapy, CBCT‐based adaptive RT, ethos‐based adaptive, MU variability, patient specific QA, plan QA

## Abstract

**Purpose:**

CBCT‐guided online‐adaptive radiotherapy (oART) systems have been made possible by using artificial intelligence and automation to substantially reduce treatment planning time during on‐couch adaptive sessions. Evaluating plans generated during an adaptive session presents significant challenges to the clinical team as the planning process gets compressed into a shorter window than offline planning. We identified MU variations up to 30% difference between the adaptive plan and the reference plan in several oART sessions that caused the clinical team to question the accuracy of the oART dose calculation. We investigated the cause of MU variation and the overall accuracy of the dose delivered when MU variations appear unnecessarily large.

**Methods:**

Dosimetric and adaptive plan data from 604 adaptive sessions of 19 patients undergoing CBCT‐guided oART were collected. The analysis included total MU per fraction, planning target volume (PTV) and organs at risk (OAR) volumes, changes in PTV‐OAR overlap, and DVH curves. Sessions with MU greater than two standard deviations from the mean were reoptimized offline, verified by an independent calculation system, and measured using a detector array.

**Results:**

MU variations relative to the reference plan were normally distributed with a mean of −1.0% and a standard deviation of 11.0%. No significant correlation was found between MU variation and anatomic changes. Offline reoptimization did not reliably reproduce either reference or on‐couch total MUs, suggesting that stochastic effects within the oART optimizer are likely causing the variations. Independent dose calculation and detector array measurements resulted in acceptable agreement with the planned dose.

**Conclusions:**

MU variations observed between oART plans were not caused by any errors within the oART workflow. Providers should refrain from using MU variability as a way to express their confidence in the treatment planning accuracy. Clinical decisions during on‐couch adaptive sessions should rely on validated secondary dose calculations to ensure optimal plan selection.

## 1 INTRODUCTION

CBCT‐guided online adaptive radiation therapy (oART) is a novel treatment technique that enables clinicians to account for interfractional anatomical variations within the patient in a way standard image‐guided techniques cannot. The rapid adoption of CBCT‐guided oART has been driven by the release of the Ethos radiotherapy platform (Varian Medical Systems, Palo Alto, CA USA). The Ethos system combines several state‐of‐the‐art technologies: artificial intelligence‐driven segmentation, deformable image registration, and a new priority‐based optimizer referred to as the Intelligent Optimization Engine (IOE).^1^ The result is a system that can deliver an adapted fraction in as little as 15 min, thereby minimizing potentially problematic intrafraction motion.^2^

The short treatment times in CBCT‐guided oART present a challenge for physicists and clinicians seeking to assess plan quality and ensure process integrity. Automation is heavily relied upon to perform on‐couch quality assurance (QA) in the form of secondary dose calculations, basic structure integrity checks, and log‐file dose reconstruction following delivery.^3^ However, the increased reliance on automation in CBCT‐guided oART can make the entire system seem like a black box. Clinicians may notice day‐to‐day variations in various areas, such as disagreements between the CBCT and synthetic CT^1^ (sCT)‐generated contours, variations in the volume and location of internal air pockets,^4^ and variations in daily monitor units (MU). Gaining an understanding of which variations may present concern for the oART workflow requires an evaluation of the magnitude and impact of these various effects.

This investigation reports on our initial experience with oART as it relates to daily variations in total MU. In our clinical practice, the MU of some adaptive plans were over 10%, even as high as 40%, different from the corresponding reference^2^ plan, raising concerns about dose accuracy of On‐Couch adaptive plans and motivating this retrospective systematic investigation. We report on the range of MU variations we have experienced with oART using the Ethos system, investigate some possible causes for daily MU variations, and assess the reproducibility of such variations within the Ethos IOE.

## 2 MATERIALS AND METHODS

### 2.1 Adaptive data set

Dosimetric data generated by the Ethos On‐Couch Session^3^ for 19 patients (604 sessions) receiving oART using static field IMRT with standard and SBRT fractionation schemes to the pelvis were retrospectively gathered from the Mobius3D database (Varian Medical Systems, Palo Alto, CA). A table with additional descriptors of the dataset can be found in Appendix 1. Mobius3D was queried for all reference, adapted, and scheduled plans. Data includes total MU per fraction, structure volumes, structure mean doses, dose to 90% of each structure (D90), and DVH curves from both the scheduled^4^ and adapted^5^ plans over all fractions treated. The dose information collected from Mobius3D were the values calculated from the Ethos On‐Couch Session. Dose calculations computed in Mobius3D were not used. Variations relative to the reference plan were computed for all plans and structure sets evaluated. Data collected from Mobius3D did not include any DICOM data. All data collection were performed in accordance with the institution's review board under IRB approval.

### 2.2 Data analysis

Adaptive plan value statistics were captured using an in‐house developed script with Panda Dataframe methods^5^ and a python script provided by Varian in the documentation library that allows access to the raw data in Mobius3D. The distribution of MU variations, PTV volume differences, and OAR volume differences were evaluated for normality using the Shapiro‐Wilk test. For the Shapiro‐Wilk test, the null hypothesis is that the distribution is a normal distribution and *p* > 0.05 fails to reject the null hypothesis. We assessed the distribution of dose per fraction, total plan MU, and IMRT modulation factor, which is defined as total plan MU divided by the prescribed dose. The plan complexity of each evaluated plan was calculated in ClearCheck (Radformation, Inc., New York, NY, v2.4.3) using the field complexity metric proposed by Younge et al.^6^ At our institution, the complexity threshold used was 0.18 mm^‐1^ as recommended by Younge et al.^7^ considering no other recommendation has been published specifically for Ethos.

### 2.3 Correlation assessment with anatomical parameters (target volume and organ‐at‐risk overlap)

It was hypothesized that MU changes relative to the reference plan may be impacted by changes in both the PTV volume and the OAR‐PTV overlap. To evaluate this hypothesis, the first 60 fractions from the first eight patients treated to completion were exported from the Ethos Treatment Planning Management System (TPMS, v1.1) and imported into Velocity (Varian Medical Systems, v4.1). This data included the DICOM CT data of the synthetic CT (sCT), daily CBCT, RT Structure set, RT Plans, and RT Dose for both scheduled and adapted fractions. Reference plan data (CT, Structure set, Plan, Dose) was also imported into Velocity for comparison. An automated script was used to evaluate Organ at Risk (OAR) overlap with the target structures for each adaptive session. The primary OARs in this cohort were the bladder and rectum. This subset of the acquired data was used to calculate Pearson correlation coefficients for (a) MU change versus PTV volume change and (b) MU change versus OAR‐PTV overlap change.

### 2.4 Planning system investigation

#### 2.4.1 Ethos on‐couch versus TPMS optimization

To investigate whether the on‐couch optimizer had higher MU variability than the offline TPMS, selected fractions with variations greater than two standard deviations (σ), calculated across all adaptive sessions and patients were brought into the Ethos TPMS to produce reoptimized plans based on the session anatomy, analogous to the adaptive plans produced by the on‐couch optimizer. The sCT and patient model from each session was imported into the TPMS as a new treatment site. A template of each patient's original reference planning directive, containing the relevant clinical objectives and their relative priorities, was created and applied to the session datasets. The on‐couch generated adaptive plan was imported to reproduce the treatment isocenter and beam geometry of the on‐couch adaptive session and reoptimized using the reference planning directive conditions. MU per beam and total session MU from each reoptimized session were recorded. These were compared against the on‐couch optimized adaptive plan.

### 2.5 Secondary calculation in Mobius3D

Independent verification of the primary IOE treatment planning system, which uses the AcurosXB algorithm (Varian Medical Systems, Palo Alto, CA) for dose computation, was performed using a secondary dose calculation in Mobius3D. This secondary check software uses the sCT generated during the adaptive session along with the plan and corresponding structure set to carry out an independent 3D convolution‐superposition dose calculation, which is then compared with the dose calculated by the primary IOE treatment planning system using global 3D gamma analysis (γ). The accepted gamma passing rate was that greater than 90% of the evaluated points must satisfy the condition *γ* < 1 for a global 3% dose difference, 3 mm distance to agreement criterion, and 10% dose threshold.^6^ A subset of patients treated with SBRT treatments was also evaluated in the Mobius3D system using a γ criterion of 3%/2mm with a 10% threshold, in accordance with our institutional protocols. A Mobius3D independent calculation and a MobiusFX (delivery log‐file analysis) are created for all treated adaptive fractions following the standard adaptive QA procedure at our institution.

### 2.6 Phantom dose verification measurements

Selected adaptive sessions with MU differences greater than two standard deviations (2σ) were selected to verify the accuracy of the delivered versus calculated doses. These sessions were exported from the Ethos TPMS and imported into Eclipse (v15.6, Varian Medical Systems). The sessions were recalculated with preset MUs on the Mobius MVP phantom (Varian Medical Systems), a homogenous virtual water phantom with seven possible positions for ion chamber measurements. The plans were then delivered on the Ethos machine and ion chamber measurements were acquired with a calibrated Exradin A1SL chamber (Standard Imaging, Madison, WI USA). The ion chamber was cross‐calibrated against a measurement on the Ethos of 100 MU delivery from a 10 cm × 10 cm open field with the chamber positioned at the isocenter. Predicted doses to the ion chamber from Eclipse were reported as the mean dose to the chamber's sensitive volume.

These same selected fractions were also delivered on a Sun Nuclear ArcCheck (Sun Nuclear, Melbourne, FL USA). Predicted doses were generated in Eclipse. Composite measurement of the adaptive session was compared using γ analysis with 3%/2mm, 10% dose threshold, and global dose settings in the SNC Patient software. Task Group 218 passing criteria were used with γ < 1 for 90% of points being a passing result, with >95% of points being preferred.^8^

## 3 RESULTS

### 3.1 MU variability

**FIGURE 1:**
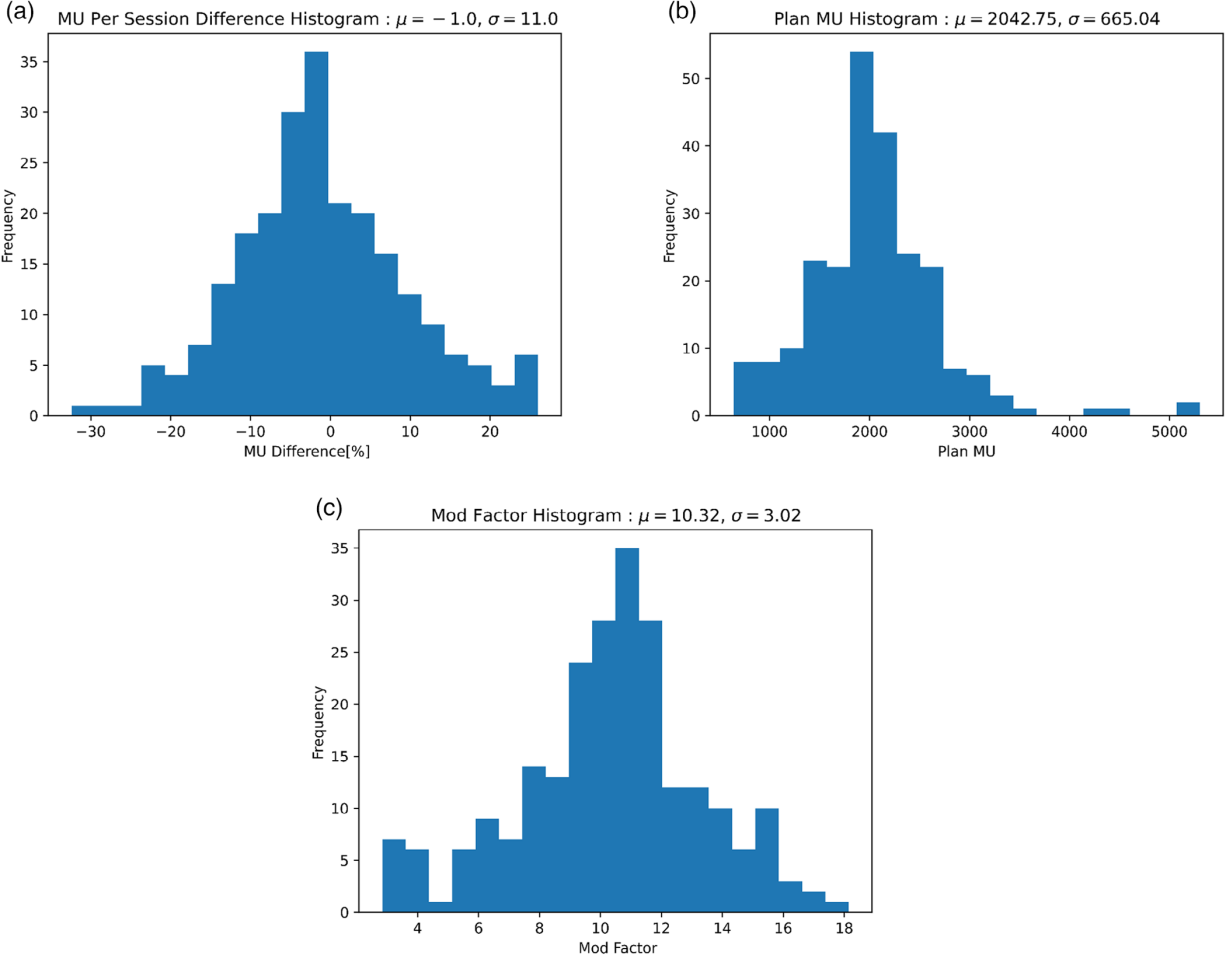
Histogram plots of (a) ensemble MU difference, (b) Plan MU, and (c) IMRT Modulation Factor (MU/cGy).

**FIGURE 2:**
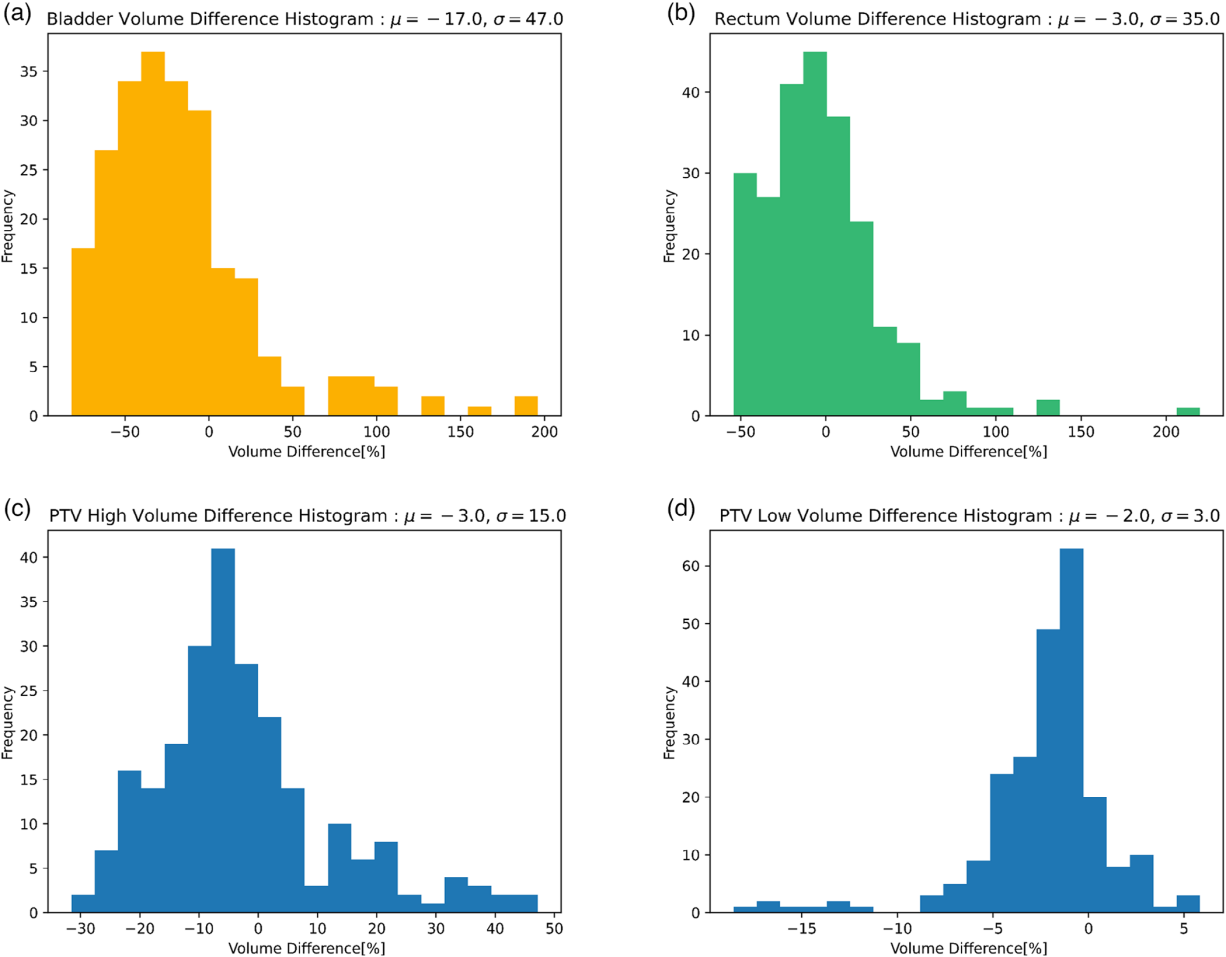
Histogram plots of ensemble volume differences for (a) Bladder, (b) Rectum, (c) PTV High, and (d) PTV Low.

Figure 1 shows that the mean modulation factor in our dataset is 10.3 and the median planned dose per fraction was 1.8 Gy. The median and mean total plan MU were 1996 and 2043 MU, respectively. We observed a normal (*p*_Shapiro‐Wilk_ = 0.119) distribution of MU variation with a mean of −1.0% and a standard deviation of 11.0%. Fourteen fractions demonstrated MU variations >2σ (22%). MU variations were assessed longitudinally for each patient over the course of treatment with OAR and PTV variations in Figure 2. For patients with multiple PTVs, the analysis was done using PTV High for the target with a higher prescription dose and PTV Low for the target with a lower prescription dose. The prescription dose for PTV High and PTV Low is reported for each patient in Appendix 1.

### 3.2 Correlation between MU changes and anatomical parameters

**FIGURE 3:**
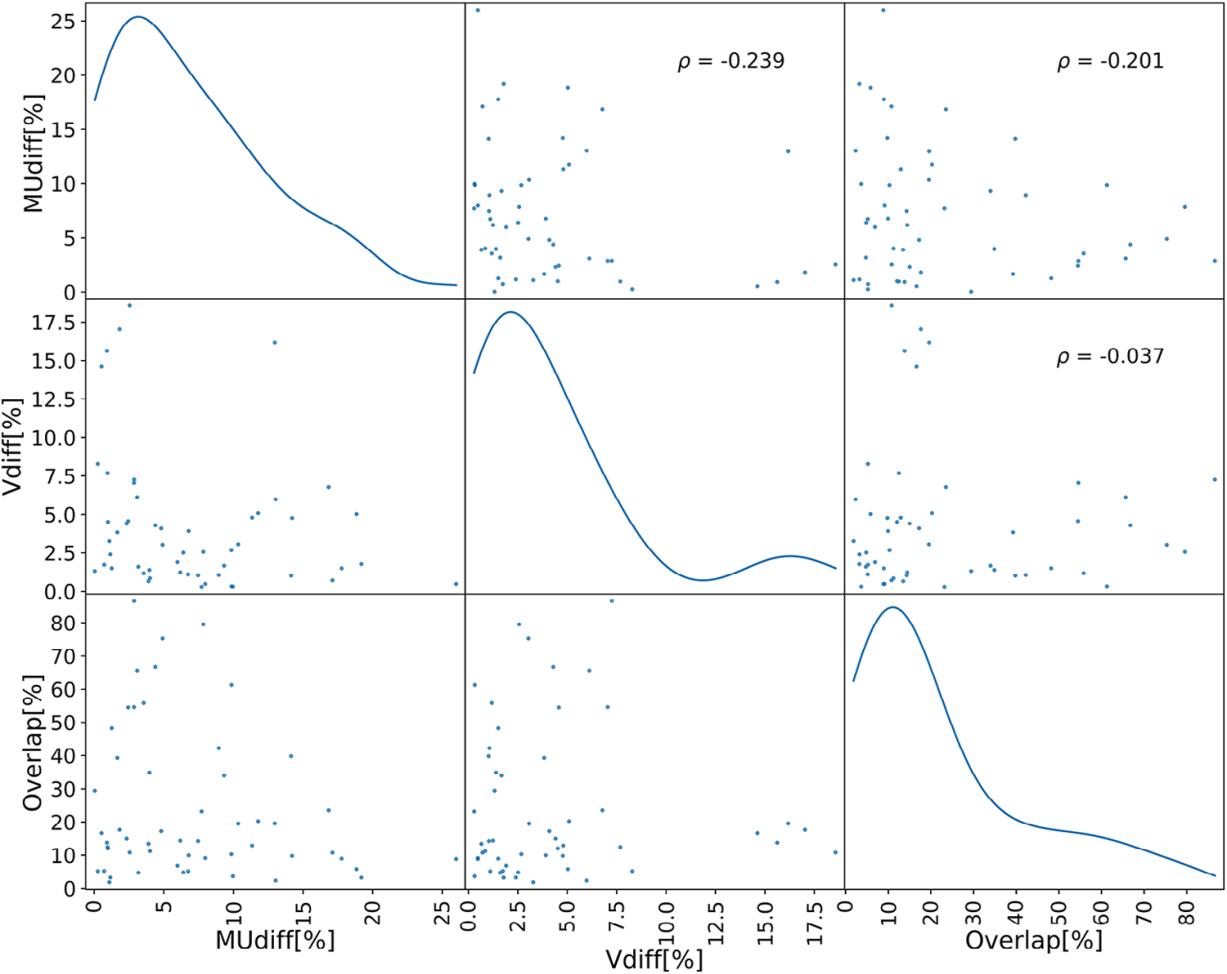
Pearson correlation plots and correlation coefficients between absolute MU differences, PTV volume differences and PTV‐OAR overlap differences relative to the reference plan. Kernel density estimation (KDE) plotted in the diagonal.

**FIGURE 4:**
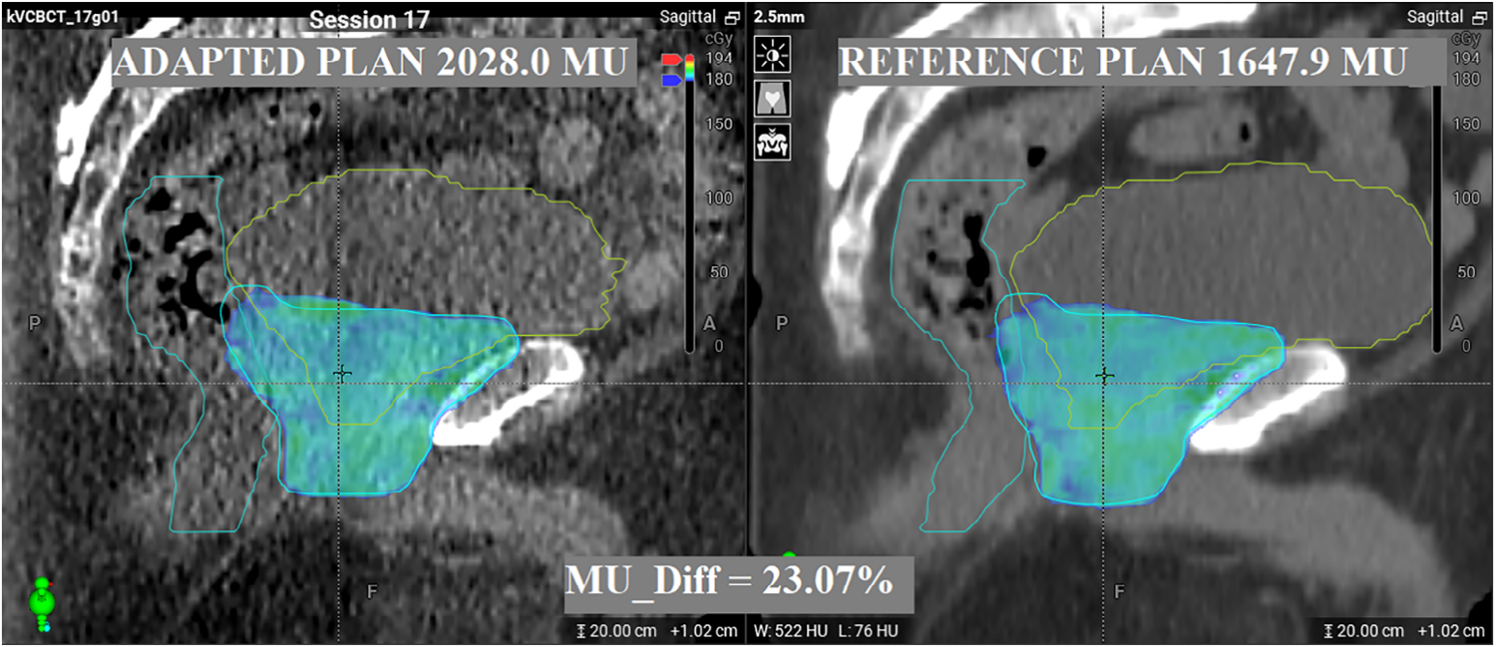
Example of a session with high MU differences at 381 MU (23.07%) showing minimal changes in PTV or OARs. PTV volume change 1.60 cc (7.54%), rectum volume change 7.74 cc (11.36%), and bladder volume change 61.60 cc (13.72%).

OAR volume variations did not demonstrate any correlation with MU variation. Pearson correlation coefficients (*ρ*) between MU difference and rectum and bladder volume differences were *ρ* = −0.04 and *ρ* = −0.03 respectively. Scatter correlation plots comparing MU variation, target volume differences, and target‐OAR overlap volume differences are shown in Figure 3, along with the Pearson correlation coefficients. No significant correlation was observed between the changes in MU relative to the reference plan and any of the parameters investigated. Figure 4 shows a visual example of MU changes that cannot be easily justified by changes in the PTV or OAR.

### 3.3 Ethos on‐couch versus TPMS optimization

**FIGURE 5:**
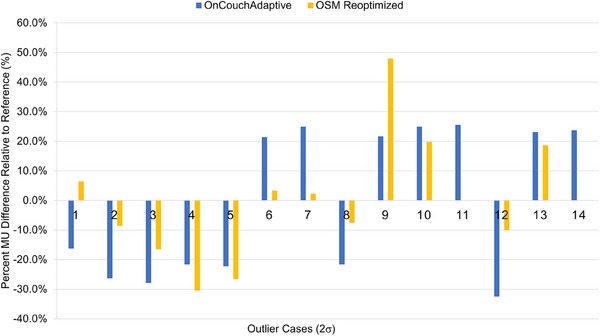
Percentual MU difference relative to the reference plan for on‐couch adaptive generated plans and on‐couch session manager reoptimized plans.

Results of reoptimizing on‐couch sessions using the TPMS with identical planning directives as seen in Figure 5 did not reliably reproduce either reference or on‐couch total MUs. The mean percent difference in reoptimized plan MUs relative to reference was 14.2 ± 19.3% while the mean percent difference between the reoptimized plans compared to the on‐couch plan was 16.9 ± 18.6%. Differences between the TPMS and the on‐couch session manager (OSM) sessions were inconsistent across the sample with some reoptimized plans returning results closer to reference plan MUs and other returning results closer to the on‐couch MU, indicating the TPMS also has high MU variability in plan optimization similar to the on‐couch optimizer.

### 3.4 Secondary dose calculation verification Mobius3D

Adaptive plans that produced a change in MU greater than 2σ relative to the reference plan were recalculated in Mobius 3D and compared to the Ethos‐generated plan as described in the methods. Plan comparison results are shown in Table 1. All recalculated plans demonstrated gamma passing rates >98% at 3%/3 mm. Four plans were also evaluated at 3%/2 mm as per our SBRT‐institutionally defined tolerance. Under the stricter SBRT criterion, passing rates greater than 98% were still observed. Plan complexity was considered high but within institutional tolerance (≤0.18).

**TABLE 1:** Secondary dose calculation and verification for adaptive plans with a change in MU >2*σ* relative to reference plan.

	ΔMU[%] >2σ	Plan complexity (adaptive)[mm^‐1^]	Independent calculation M3D γ [3%/3][%] or SBRT γ (3%/2 mm)	Ion chamber verification ($\frac{D_{\mathrm{measured}}-D_{\mathrm{calculated}}}{D_{\mathrm{calculated}}})$[%]	Diode array verification γ_3%/2mm_ <1[%]
1	−16.23	0.15	98.8	1.45	96.8
2	−26.33	0.14	100 (99.3)	0.97	96.9
3	−27.84	0.15	100 (99.4)	0.29	99
4	−21.63	0.15	100 (99.7)	0.87	99.3
5	−22.19	0.15	100 (99.6)	1.14	96.5
6	21.37	0.17	99.0	1.16	95.9
7	24.92	0.17	99.2	1.26	94.7
8	−21.62	0.15	100	0.41	97.5
9	21.67	0.16	99.4	1.43	93.3
10	24.95	0.17	99.5	1.72	94.8
11	25.52	0.17	99.3	1.94	92.4
12	−32.42	0.12	100	1.49	98.7
13	23.07	0.18	99.8	1.90	95.7
14	23.69	0.17	99.7	2.15	95.4

### 3.5 Dose calculation verification

Adaptive plans that produced a change in MU greater than 2σ relative to the reference plan were delivered to a diode array and to an ion chamber as described in the methods. Verification measurements are shown Table 1. All ArcCheck measurements demonstrated gamma passing rates >90% at 3%/2mm. All ion chamber measurements were within 2.5% of the calculated dose.

## 4 DISCUSSION

Ethos oART treatment planning optimizer was previously reported to yield higher MU than the Eclipse treatment planning optimizer from the same vendor.^9^ However, a new observation of MU variability in Ethos on adaptive plans was made in our clinical practice. This investigation details the range of MU variability found in oART using a novel priority‐based treatment planning optimizer designed for use in CBCT‐guided oART. We have found that on‐couch MU variation for oART follow a normal distribution with a mean of roughly 1.0% and a standard deviation of 11.0%. However, the range of this distribution can reach extremes of ± 30%. We have investigated the reproducibility of these variations within the Ethos TPMS, and if these changes are driven by variations in the daily anatomy of the patient. Ethos TPMS was found to have similar MU variability or lack of reproducibility. We have also found no correlation between anatomic variation and MU variations. The accuracy of dose calculation was verified with ion chamber and ArcCheck measurements.

### 4.1 Ethos TPMS reproducibility

The Ethos TPMS uses a novel priority‐based optimizer to generate treatment plans as described by Archambault et al.^1^ The Ethos oART platform utilizes the IOE both for reference plan calculation and on‐couch optimization. Varian Ethos documentation states that both the TPMS and on‐couch planning sessions utilize identical algorithms for optimization, plan generation, and dose calculation. Therefore, the differences between daily on‐couch plans and reference plans can only be caused by variations in patient anatomy relative to reference or to stochastic effects within the IOE itself. Stochastic effects are known to be a part of the IOE^9^ as one of the improvements that drove Ethos v1.1 was a reduction in variations of plan quality with successive iterations using identical patient geometries. However, the claims of reductions in variations between v1.0 and v1.1 only relate to plan quality as measured by the entered clinical objectives, not other metrics related to the plan such as total MU, IMRT modulation factor, or plan complexity scores.

Reoptimization of identical geometries using the emulator software demonstrated that the MU variation trended in the same direction as that observed on‐couch, with one exception in the sample, but that the magnitude of that variation was not predictable. This suggests that there is some causal relationship between daily anatomy and plan complexity, but the fact that the magnitudes of variations were not reliably reproduced suggests that relationship is influenced by some randomness from the optimizer.^9^

### 4.2 Effects of modulation

Increased MU variability leads to <1% overall change in total MU delivered on average but can create large variations in beam‐on time on an inter‐fractional basis. Treatment plans with large decreases in MU are in many ways more desirable; they represent a class of solutions with decreased beam‐on time and less complex fluence patterns that allow the patient geometry the least likelihood to change during treatment delivery. However, large increases in MU relate directly to our IMRT modulation factor and plan complexity metrics, leading to longer irradiation times with highly complex fluence distributions. The compounding nature of longer beam‐on time and increased plan complexity raises the likelihood of the interplay effect as described by Seco et al.^10^ While the Ethos delivery system has been shown to be capable of treating patients adaptively within standard treatment appointment time slots, the system can only rely on surface tracking to verify the patient's positioning after CBCT acquisition, unlike its correlates in the MR‐linac space. These surface tracking systems cannot capture changes in internal anatomy nor provide any methods for the user to assess the dosimetric impact of intrafractional anatomic variation that occurs following the acquisition of the daily CBCT. Increasing plan complexity and time‐on‐couch are both enemies of the overall goal of adaptive therapy – to maximally spare normal tissues and deliver the highest dose to target structures as accurately as possible throughout treatment.

High MU variability also can cause concerns for the clinical team during the on‐couch adaptive sessions. Large changes in MU relative to the reference plan may just be a property of the IOE, but oftentimes MU consistency provides the clinical team with confidence that the newly generated treatment plan is safe to deliver. Deviations >20% can lead to questions during the treatment session about the accuracy of the overall dose calculation, or wonderings as to why the changes are so drastic one day compared to other days. As a result of the lack of apparent answers as to the origin of these large variations, combined with the heightened time pressures intrinsic to the nature of oART, the clinical team may choose a suboptimal plan because it seems like the safer choice. Our investigation indicates that the dose calculation is working well within pre‐defined thresholds and that large MU variations are just a function of the stochastic nature of this system. Clinical decisions should be justified using reliable data from the on‐couch planning system and a validated secondary dose calculation to ensure that the most optimal plan is being chosen daily.

### 4.3 Dose calculation accuracy

Treatment plans with MU variation >2σ were measured both using an ArcCheck phantom and a cross‐calibrated ion chamber in a homogenous phantom. The ArcCheck measurements were acquired to validate the accuracy of calculation across the entire treatment field. The ion chamber measurements were acquired to assess the accuracy of the cumulative dose in a well‐defined region. Both sets of measurements were well within our institutional tolerances of 3% dose difference for ion chamber and array measurements. These results are consistent with two prior studies that reported retrospective patient‐specific QA results for patients undergoing adaptive radiotherapy.^11^, ^12^ Our study demonstrates that the IOE and AcurosXB algorithms driving the oART session can be trusted, even for plans where large variations from the reference exist.

## 5 CONCLUSIONS

We have observed and investigated the causes for variation in MU during oART using the Ethos ART platform. MU variation correlates well with IMRT modulation factor and plan complexity metrics but does not correlate with any patient anatomy considerations investigated in this work such as target or OAR volume or target/OAR overlap. Based on our investigation, it appears that MU variations are normally distributed about 1.0% difference with a standard deviation of 11.0%. These values should be verified by other Ethos users. Dose distributions calculated from the Ethos platform were validated against the Mobius3D secondary dose calculation system and confirmed with measurement using an ArcCheck and ion chamber for fractions with variations >2σ from the reference plan. MU variations will exist within oART plans but are likely not caused by any errors within the oART workflow. Providers can incorporate MU variations into their assessment of oART plan quality as it pertains to intrafractional uncertainty but should refrain from using this as a way to express their confidence in the treatment planning accuracy.

## AUTHOR CONTRIBUTIONS

Sean Tanny and Olga M Dona Lemus designed the project, acquired, and analyzed the data, and wrote the manuscript. Sean Tanny and Nicholas Sperling developed data the acquisition tool and revised the manuscript. Joshua Wancura, Matthew Webster, Hyunuk Jung, Yuwei Zhou, Fiona Li, Jihyung Yoon, Alexander Podgorsak and made significant contribution to the design of the work and revised the manuscript. Dandan Zheng provided mentorship and resources needed for the project, contributed to the conception and design of the work, and revised the manuscript. All authors approved the submitted draft and agreed to be accountable for all aspects of this work.

## CONFLICT OF INTEREST STATEMENT

The authors certify that they have NO affiliations with or involvement in any organization or entity with any financial interest (such as honoraria; educational grants; participation in speakers’ bureaus; membership, employment, consultancies, stock ownership, or other equity interest; and expert testimony or patent‐licensing arrangements), or non‐financial interest (such as personal or professional relationships, affiliations, knowledge or beliefs) in the subject matter or materials discussed in this manuscript.

## Supporting information

Supporting Information

## Footnotes

1 The Synthetic CT (sCT) is generated for dose calculation purposes using deformable registration between the planning CT and the daily CBCT.

2 The reference plan refers to the initial plan created based on the simulation CT.

3 On Couch‐Session refers to the treatment session. For the on couch‐session an image is acquired, contours generated, scheduled and adaptive plans are calculated, and the selected treatment is delivered.

4 Scheduled plan refers to the reference plan calculated on the synthetic CT created from the on couch‐session image (CBCT).

5 Adapted plan refers to a plan reoptimized based on on‐couch session contours and calculated on the synthetic CT created from the on‐couch session image (CBCT).

## References

[1] Archambault Y , Boylan C , Bullock D , et al. Making on‐line adaptive radiotherapy possible using artificial intelligence and machine learning for efficient daily re‐planning. Med Phys Intl J. 2020;8(2):77‐86.

[2] Zwart LGM , Ong F , ten Asbroek LA , et al. Cone‐beam computed tomography‐guided online adaptive radiotherapy is feasible for prostate cancer patients. Phys Imaging Radiat Oncol. 2022;22:98‐103. 10.1016/j.phro.2022.04.009 35602545 PMC9115122

[3] Byrne M , Archibald‐Heeren B , Hu Y , et al. Varian ethos online adaptive radiotherapy for prostate cancer: Early results of contouring accuracy, treatment plan quality, and treatment time. J Appl Clin Med Phys. 2022;23(1):e13479. 10.1002/acm2.13479 34846098 PMC8803282

[4] Lemus OMD , Tanny S , Cummings M , et al. Influence of air mapping errors on the dosimetric accuracy of prostate CBCT‐guided online adaptive radiation therapy. J Appl Clin Med Phys. 2023;24:e14057. 10.1002/acm2.14057 37276082 PMC10562036

[5] McKinney W . Data Structures for Statistical Computing in Python. In: Proceedings of the 9th Python in Science Conference. 2010;56‐61. 10.25080/Majora-92bf1922-00a

[6] Younge KC , Matuszak MM , Moran JM , McShan DL , Fraass BA , Roberts DA . Penalization of aperture complexity in inversely planned volumetric modulated arc therapy. Med Phys. 2012;39(11):7160‐7170. 10.1118/1.4762566 23127107 PMC3505204

[7] Younge KC , Roberts D , Janes LA , Anderson C , Moran JM , Matuszak MM . Predicting deliverability of volumetric‐modulated arc therapy (VMAT) plans using aperture complexity analysis. J Appl Clin Med Phys. 2016;17(4):124‐131. 10.1120/jacmp.v17i4.6241 PMC5345484 27455504

[8] Miften M , Olch A , Mihailidis D , et al. Tolerance limits and methodologies for IMRT measurement‐based verification QA : *recommendations of AAPM/Task Group No. 218* . Med Phys. 2018;45(4):e53‐e83. 10.1002/mp.12810 29443390

[9] Pokharel S , Pacheco A , Tanner S . Assessment of efficacy in automated plan generation for varian ethos intelligent optimization engine. J Appl Clin Med Phys. 2022;23(4):e13539. 10.1002/acm2.13539 35084090 PMC8992949

[10] Seco J , Sharp GC , Turcotte J , Gierga D , Bortfeld T , Paganetti H . Effects of organ motion on IMRT treatments with segments of few monitor units. Med Phys. 2007;34(3):923‐934. 10.1118/1.2436972 17441238 PMC2034283

[11] Zhao X , Stanley DN , Cardenas CE , Harms J , Popple RA . Do we need patient‐specific QA for adaptively generated plans? Retrospective evaluation of delivered online adaptive treatment plans on varian ethos. J Appl Clin Med Phys. 2023;24(2):e13876. 10.1002/acm2.13876 36560887 PMC9924122

[12] Shen C , Chen L , Zhong X , et al. Clinical experience on patient‐specific quality assurance for CBCT‐based online adaptive treatment plan. J Appl Clin Med Phys. 2023;24(4):e13918. 10.1002/acm2.13918 36729373 PMC10113688

