# Supplementary material for: MU variability in CBCT‐guided online adaptive radiation therapy
Source: J Appl Clin Med Phys. 2024 Jun 19;25(9):e14440. doi: 10.1002/acm2.14440 (PMC11492364; doi:10.1002/acm2.14440)
Supplement: Supplementary file 1 — Supporting Information [file ACM2-25-e14440-s001.docx]

Table 1. Reference plan descriptors for the adaptive dataset

| Patient Index | Site | Phase | Dose per Fraction | Number of Fractions | Total Dose | Reference plan MU | PTV High; PTV Low |
| --- | --- | --- | --- | --- | --- | --- | --- |
| NY-167-00001 | Rectum | PH1 | 200 | 25 | 5000 | 3259.7 | PTV5000cGy; PTV4500cGY |
|  |  | PH2 | 200 | 2 | 400 | 1100.2 | PTV5400cGy |
| NY-167-00002 | Prostate | PH1 | 180 | 25 | 4500 | 2018.5 | PTV4500cGy |
|  |  | PH2 | 180 | 13 | 2340 | 711.7 | PTV6840cGy |
| NY-167-00003 | Prostate | PH1 | 180 | 25 | 4500 | 1964.8 | PTV4500cGy |
|  |  | PH2 | 180 | 13 | 2340 | 1484.2 | PTV6840cGy |
| NY-167-00004 | Prostate | Single Course | 725 | 5 | 3625 | 5460.5 | PTV3625cGy |
| NY-167-00005 | Prostate | Single Course | 180 | 25 | 4500 | 1647.9 | PTV4500cGy |
| NY-167-00006 | Rectum | PH1 | 200 | 25 | 5000 | 2111.6 | PTV5000cGy; PTV4500cGy |
|  |  | PH2 | 180 | 2 | 360 | 762 | PTV5360cGy |
| NY-167-00007 | Rectum | PH1 | 200 | 25 | 5000 | 2107 | PTV5000cGy; PTV4500cGy |
|  |  | PH2 | 180 | 2 | 360 | 771.3 | PTV5360cGy |
| NY-167-00008 | Rectum | PH1 | 200 | 25 | 5000 | 2111.6 | PTV5000cGy; PTV4500cGy |
|  |  | PH2 | 180 | 2 | 360 | 762 | PTV5360cGy |
| NY-167-00009 | Prostate | PH1 | 180 | 25 | 4500 | 1647.9 | PTV4500cGy |
|  |  | PH2 | 180 | 13 | 2340 | 1007.1 | PTV6840cGy |
| NY-167-00010 | Prostate | PH1 | 250 | 25 | 6250 | 2505.5 | PTV6250cGy |
|  |  | PH2 | 250 | 3 | 750 | 1744.4 | PTV7000cGy |
| NY-167-00011 | Bladder | PH1 | 200 | 25 | 5000 | 2250.6 | PTV5000cGy; PTV4500cGY |
|  |  | PH2 | 200 | 7 | 1400 | 1573.1 | PTV6400cGy |
| NY-167-00012 | Prostate | PH1 | 180 | 25 | 4500 | 2135.7 | PTV4500cGy |
|  |  | PH2 | 180 | 13 | 2340 | 1547.6 | PTV6840cGy |
| NY-167-00013 | Prostate | PH1 | 180 | 25 | 4500 | 2807.6 | PTV4500cGy |
|  |  | PH2 | 180 | 13 | 2340 | 1734 | PTV6840cGy |
| NY-167-00014 | Prostate | PH1 | 220 | 27 | 5940 | 2583.5 | PTV5940cGy; PTV5400, PTV4860cGy |
|  |  | PH2 | 200 | 12 | 2400 | 1125.1 | PV7800cGy |
| NY-167-00015 | Prostate | PH1 | 180 | 25 | 4500 | 2038.5 | PTV4500cGy |
|  |  | PH2 | 180 | 13 | 2340 | 1645.2 | PTV6840cGy |
| NY-167-00016 | Prostate | PH1 | 180 | 25 | 4500 | 2277 | PTV4500cGy |
|  |  | PH2 | 180 | 13 | 2340 | 1646.3 | PTV6840cGy |
| NY-167-00017 | Prostate | PH1 | 180 | 27 | 4860 | 2248.8 | PTV4860cGy |
|  |  | PH2 | 180 | 11 | 1980 | 1828 | PTV6840cGy |
| NY-167-00018 | Prostate | PH1 | 200 | 27 | 5400 | 1994 | PTV5400cGy; PTV4860cGy |
|  |  | PH2 | 180 | 11 | 1980 | 1245.4 | PTV6840cGy |
| NY-167-00019 | Prostate | PH1 | 180 | 25 | 4500 | 2263.6 | PTV4500cGy |
